# Supplementary material for: HIF-1α and HIF-2α Differently Regulate the Radiation Sensitivity of NSCLC Cells
Source: Cells. 2019 Jan 12;8(1):45. doi: 10.3390/cells8010045 (PMC6356534; doi:10.3390/cells8010045)

SUPPLEMENTARY TABLE 1: Antibodies used in this study

Antibody	Dilution	Vendor
HIF-1α	1:500	BD Biosciences
HIF-2α	1:1000	Novus Biologicals
HIF-1β	1:1000	BD Biosciences
Lamin-A	1:1000	Sigma-Aldrich
pATM	1:1000	Abcam
pCHK2	1:1000	Bioke
CHK2	1:1000	Bioke

SUPPLEMENTARY TABLE 2: Primers used in this study

Genes	Primers	Sequence
CAIX	Forward	CATCCTAGCCCTGGTTTTTGG
CAIX	Reverse	GCTCACACCCCCTTTGGTT
GLUT1	Forward	GATTGGCTCCTTCTCTGTGG
GLUT1	Reverse	TCAAAGGACTTGCCCAGTTT
CITED2	Forward	ACGCCTTCAACGCCCTAATGG
CITED2	Reverse	ATGCCTGATGCCGCTCGTG
TWIST1	Forward	GGAGTCCGCAGTCTTACGAG
TWIST1	Reverse	TCTGGAGGACCTGGTAGAGG
MCT1	Forward	TATGGTGGAGGTCCTATCAGC
MCT1	Reverse	TGTACGGTGTTACAGAAAGAAGC
MCT4	Forward	TTTGTGCTTTACGCCGTGG
MCT4	Reverse	CTGAAGAGGTAGACGGAGTAGG
HIF-2α	Forward	TGCTCCCACGGCCTGTAC
HIF-2α	Reverse	TTGTCACACCTATGGCATATCACA
HPRT	Forward	TATTGTAATGACCAGTCAACAG
HPRT	Reverse	GGTCCTTTTCACCAGCAAG

Supplementary Figure 1

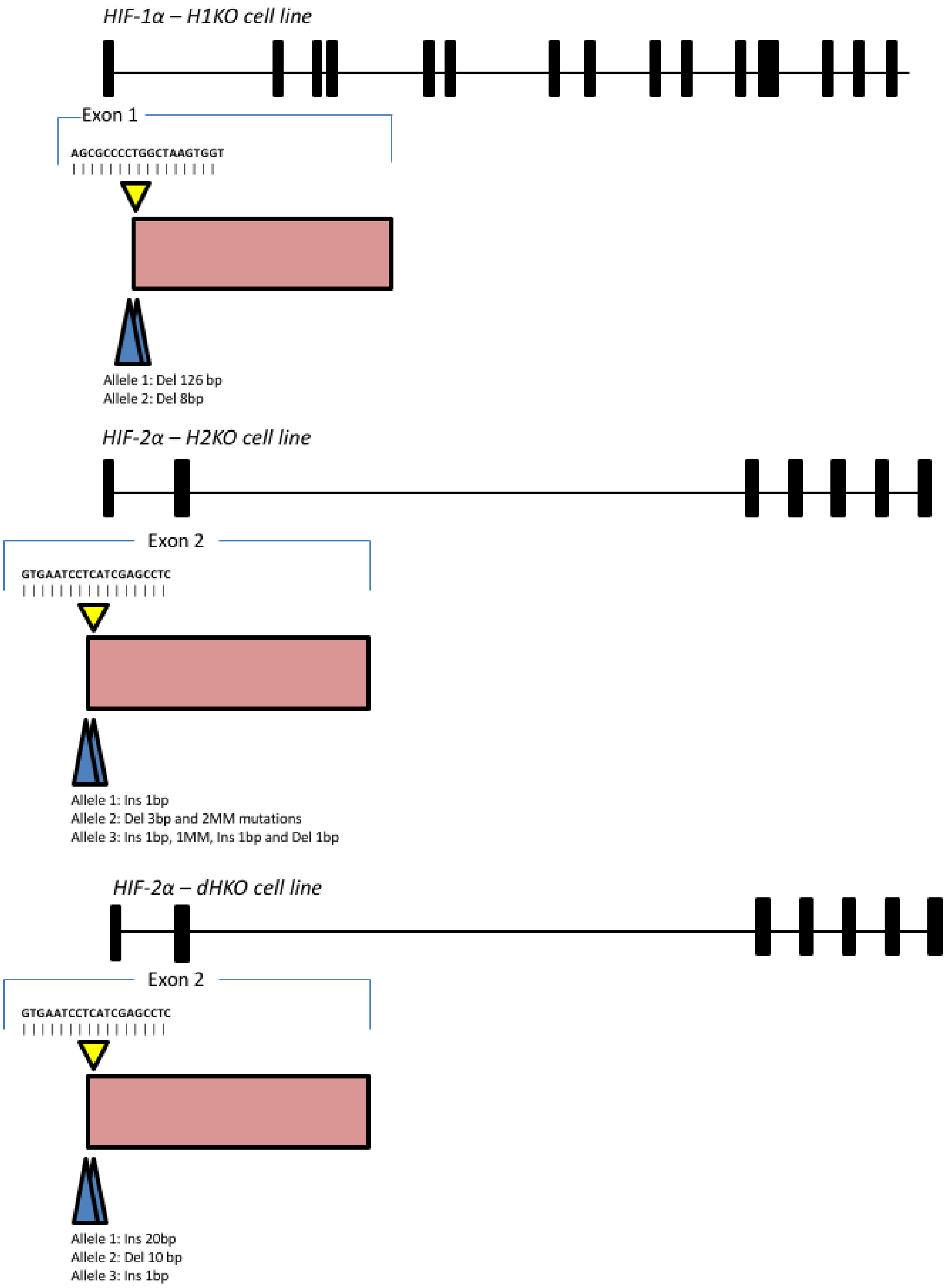

Supplementary Figure 2

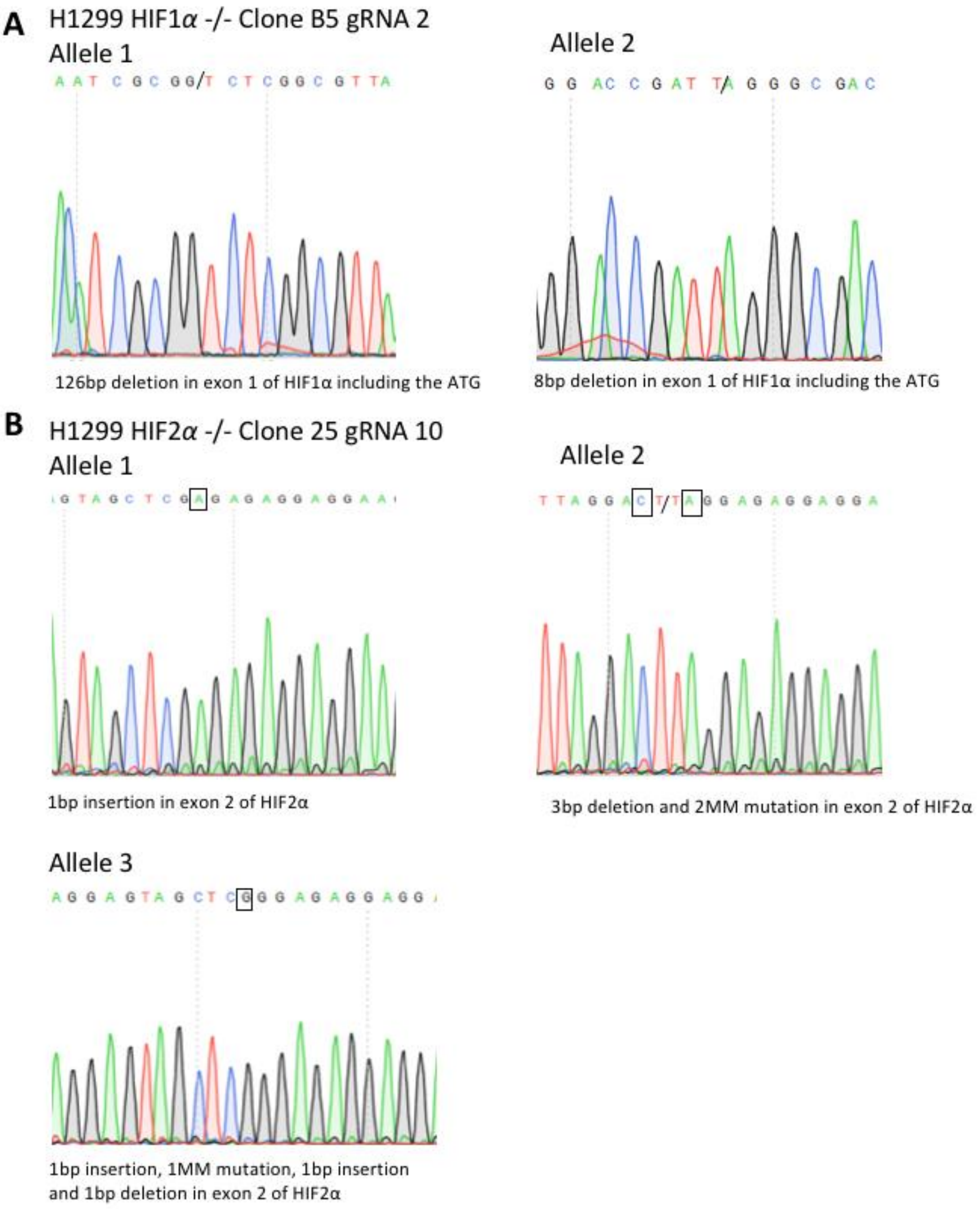

C H1299 HIF2α -/- Clone 36.103 gRNA 10

Allele 1

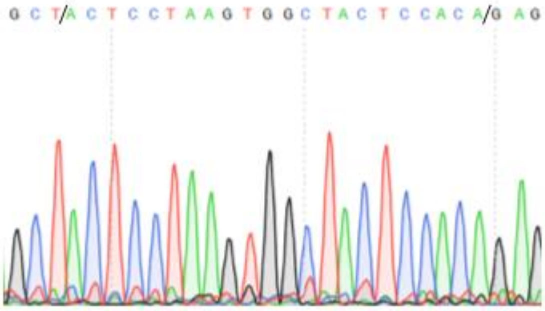

20bp insertion in exon 2 of HIF2α

Allele 2

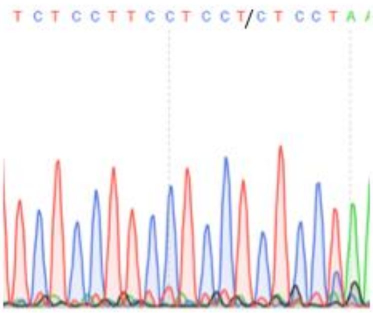

10bp deletion in exon 2 of HIF2α

Allele 3

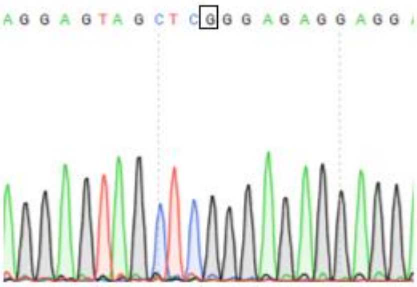

1bp insertion in exon 2 of HIF2α

Supplementary Figure 3

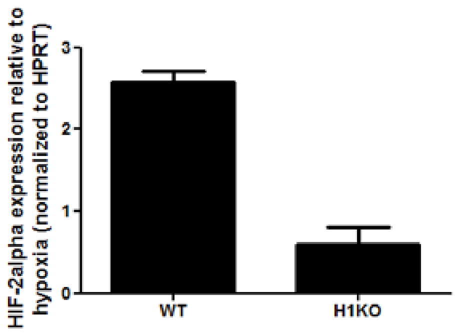

Supplementary Figure 4

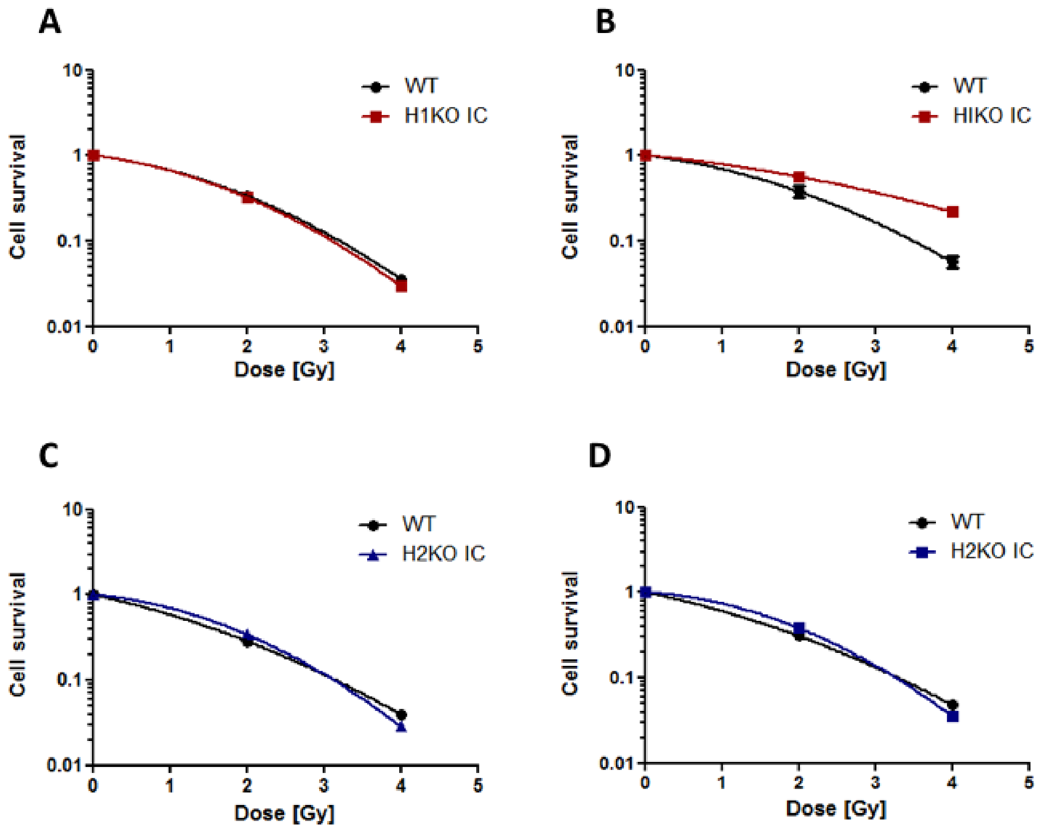

Supplementary Figure 5

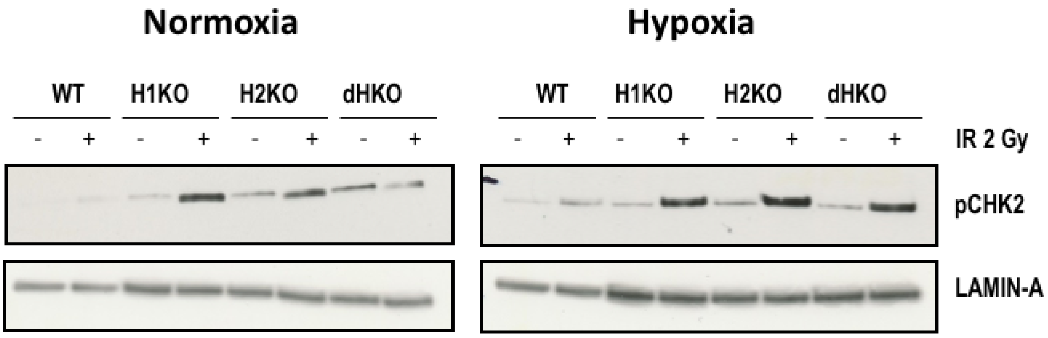

Supplementary Figure 6

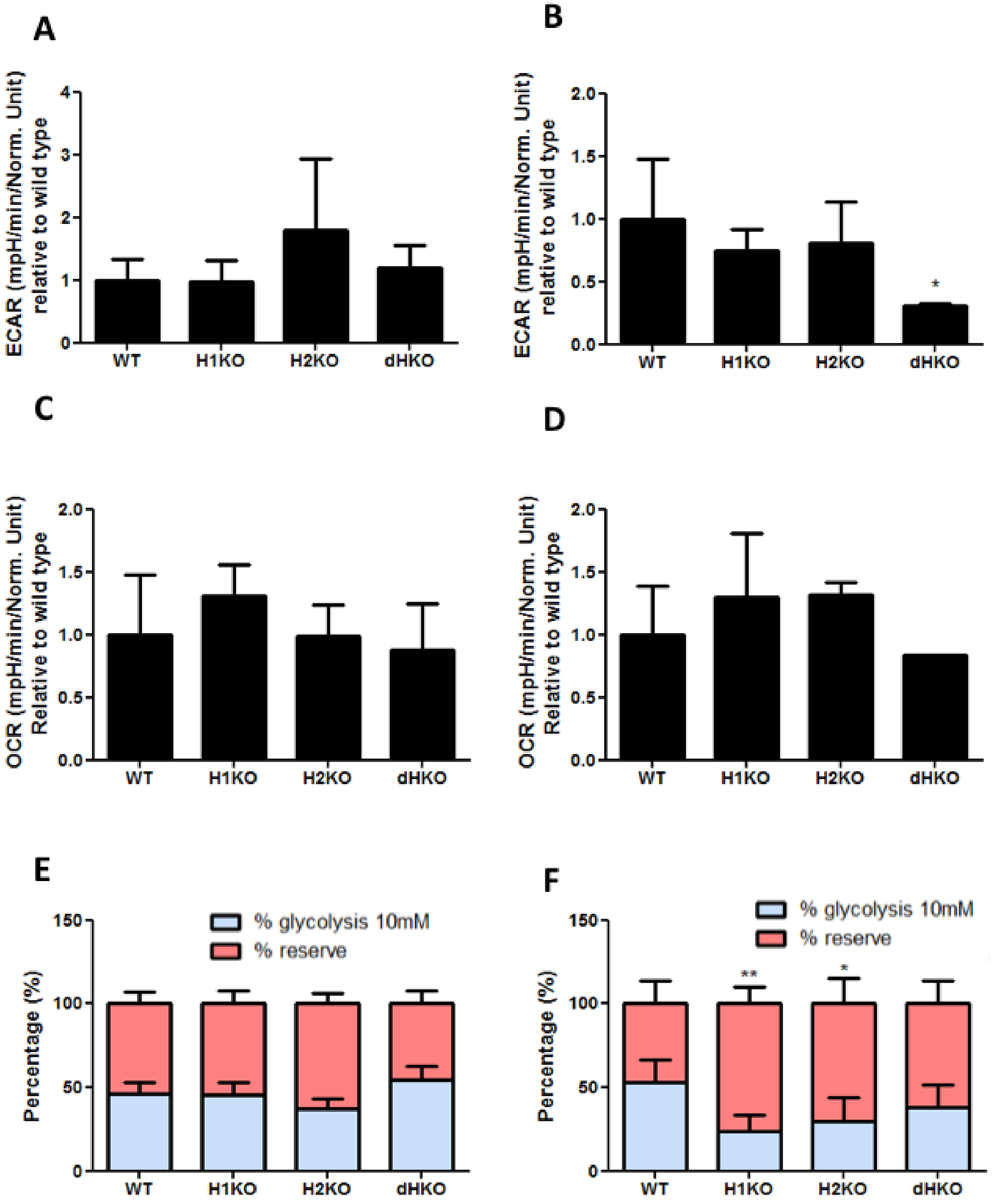

Supplement: Supplementary file 1 [file cells-08-00045-s001.pdf]
